# Cutting-Edge Vibration Sensor Morphologically Configured by Mimicking a Tactile Cutaneous Receptor Using Magnetic-Responsive Hybrid Fluid (HF)

**DOI:** 10.3390/s25113366

**Published:** 2025-05-27

**Authors:** Kunio Shimada

**Affiliations:** Faculty of Symbiotic Systems Sciences, Fukushima University, 1 Kanayagawa, Fukushima 960-1296, Japan; shimadakun@sss.fukushima-u.ac.jp; Tel.: +81-24-548-5214

**Keywords:** vibration sensor, cutaneous receptors, mimesis, rubber, electrolytic polymerization, hybrid fluid (HF), robotics, magnetic cluster

## Abstract

Vibration sensors are important in many engineering fields, including industry, surgery, space, and mechanics, such as for remote and autonomous driving. We propose a novel, cutting-edge vibratory sensor that mimics human tactile receptors, with a configuration different from current sensors such as strain gauges and piezo materials. The basic principle involves the perception of vibration via touch, with a cutaneous mechanoreceptor that is sensitive to vibration. We investigated the characteristics of the proposed vibratory sensor, in which the mechanoreceptor was covered either in hard rubber (such as silicon oil) or soft rubber (such as urethane), for both low- and high-frequency ranges. The fabricated sensor is based on piezoelectricity with a built-in voltage. It senses applied vibration by means of hairs in the sensor and the hardness of the outer cover. We also investigated two proposed parameters: the sensor response time to stimuli to the vibration aiding the equivalent firing rate (e.f.r.) and the gauge factor (*GF*_,*pe*_) proposed as treated in piezo-resistivity. The evaluation with the parameters was effective in designing a sensor based on piezoelectricity. These parameters were enhanced by the hairs in the sensor and the hardness of the outer cover. Our results were helpful for designing the present novel vibratory sensor.

## 1. Introduction

The feasibility of fabricating artificial sensors that mimic biological features of the human body, such as cutaneous mechanoreceptors, for use in various engineering fields, including industry, surgery, space, and mechanics [1,2] is an ongoing area of research [3,4,5,6]. The idea is to fabricate mechanoreceptors based on the fundamental response of humans to tactile sensations [7]. Other physical applications of mechanoreceptors involving the performance of vibratory sensing based on bioelectronics have been well demonstrated [8,9,10,11,12,13]. The present study proposes a cutting-edge artificial vibration sensor.

In current vibration sensors, the morphology of the sensory system involves piezo-resistivity or piezo-electricity. The former requires an extraneous power source for the output of the electric signal to be measured by the artificial sensor, generated by the mobile ionized electrons and holes, to induce a built-in current. The mobilization of the current can be measured by the resistance. Meanwhile, the latter does not need an extraneous power source, as it is self-powered with the measured electric signal as a built-in voltage generated by the static ions. Although the former can be created using a commonly known piezo material, it has the disadvantage of requiring the necessary power source. The latter, on the other hand, is convenient in that it does not require a power source. The self-powered sensor is also practicable with the tribo-electricity [14]. In the present study, we develop a vibration sensor based on piezo-electricity with intelligence similar to the sensory systems of human mechanoreceptors.

## 2. Materials

In the current research, artificial receptors mimicking tactile sensations on the skin have been addressed as e-skin [15], using strain gauges [16] and matrix networks [17], for the development of auditory [18,19], equilibrium [20,21], gustatory [22,23,24], and olfactory [25,26,27] sensor technology. The author in the present paper has grappled with the five human senses to detect sensation functioning in the human skin, ear, tongue, nose, and eye through their other research. Utilizing the findings, we can fabricate many types of mimicked mechanoreceptors made of soft, elastic rubber with various types of electric circuits using our proposed electrolytic polymerization.

In the present study, we adopt the same basic style of morphological fabrication to create an artificial mechanoreceptor mimicking the human cutaneous receptor. The procedure of the fabrication and the components of the materials are summarized as follows.

HF rubber 1 involves a hair-thin wire connected to the body of the mechanoreceptor fabricated with HF rubbers 2–4. HF rubbers 2 serve as the condenser, HF rubber 3, part of the outer cover of the sensor, is adhered to HF rubber 1, using HF rubber 4 as the adhesive. The ingredients of HF rubbers 1–4 are presented in Table 1.

We first produced HF with the following components: 3 g water, 3 g kerosene, 3 g silicon oil (KF96 with 1-cSt viscosity, which would solidify Q; Shin-Etsu Chemical Co., Ltd., Tokyo, Japan), 21 g PVA (Diax Co., Ltd., Fukuoka, Japan), 3 g Fe_3_O_4_ particle (Fujifilm Wako Chemicals Co., Ltd., Osaka, Japan), 3 g Fe particles (M300, about 50 μm particles; Kyowa Pure Chemical Co., Ltd., Tokyo, Japan), and 4 g sodium hexadecyl sulfate aqueous (Fujifilm Wako Chemicals Co., Ltd., Osaka, Japan) solution as a surfactant. All ingredients were mixed with an agitator under air evacuation. In the next phase, we prepared HF rubbers 1–4: HF rubbers 1 and 4 are produced in a liquid state via mixing; HF rubbers 2 and 3 are solidified with electrolytic polymerization, which has been proposed as an easy solidification technique regardless of diene and non-diene rubbers without using vulcanization via sulfur under an electric field. Here, carbonyl Ni powder (No. 123, Yamaishi Co., Ltd., Noda, Japan) had μm-order particles with bumps on the surface. Because TiO_2_ (anatase type, Fujifilm Wako Chemical Co., Ltd., Osaka, Japan) is a typical body for electron transfer, we made the HF rubber more conductive by compounding it with TiO_2_.

The electrolytic polymerization technique for HF rubber is a state-of-the-art production of rubber for the artificial mechanoreceptor and can be categorized as follows:

(a) Solidification
(b) Creation of built-in voltage and current
(c) Production of porous rubber, and infiltration with a liquid
(d) Adhesion of a rubber to metal

In terms of the (a) above, the molecules of the rubber are crosslinked, a unique technique of rubber solidification, in contrast to the ordinary vulcanization with sulfur.

As for (b), this refers to both the built-in voltage and the built-in current for piezo-electricity and piezo-resistivity, respectively.

For (c), we make a rubber permeable through electrolytic polymerization using metallic hydrate (Na_2_WO_4_·2H_2_O is optimal). The permeable rubber might serve as a filter, although any liquid can be infiltrated into the rubber by vacuum evacuation, which is expected to make it effective for many engineering applications

In terms of (d), we can adhere a rubber to any metal through electrolytic polymerization using a metallic hydrate (again, Na_2_WO_4_·2H_2_O is optimal). This technique is significant for the production of a sensor, because the electric wires must be adhered to the sensor as electrodes for measuring voltage. Therefore, extraneous adhesive conjugated between the rubber and metal is unnecessary.

**Figure 1:**
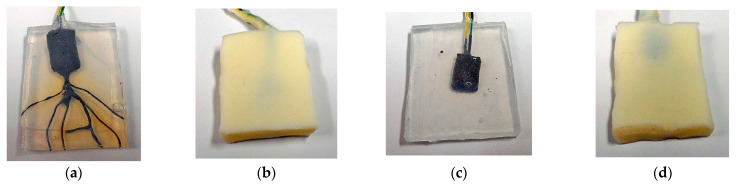
Images of fabricated sensors: (**a**) type A (with hairs, outer Q-rubber cover, dimensions 28 × 36 m × 4.5 mm thick); (**b**) type B (without hairs, outer U-rubber cover, 27 mm × 34 mm × 9 mm thick); (**c**) type C (without hairs, outer Q-rubber cover, 29 mm × 35 mm × 4.5 mm thick); (**d**) type D (with hairs, outer U-rubber cover, 26 mm × 35 mm × 7 mm thick).

Finally, we could fabricate the artificial mechanoreceptor. The fabricated mechanoreceptor was embedded in an outer cover of silicone oil (Q) rubber (Q-rubber, solidified with silicon oil (KE1300T, Shin-Etsu Chemical Co., Ltd., Tokyo, Japan)) or urethane (U) rubber (U-rubber, solidified with U-latex (HM-01 series, Exseal Co. Ltd., Gifu, Japan)) as shown in Figure 1a–d. The outer cover, rectangular-shaped for convenient installation, protects the device from effects such as deterioration due to ambient conditions. We tested the effect of the different degrees of softness of the outer cover. We also fabricated the mechanoreceptor without the hairs to investigate whether the hairs detect the vibrations by comparing the mechanoreceptors with and without the hairs.

## 3. Experimental Procedure

**Figure 2:**
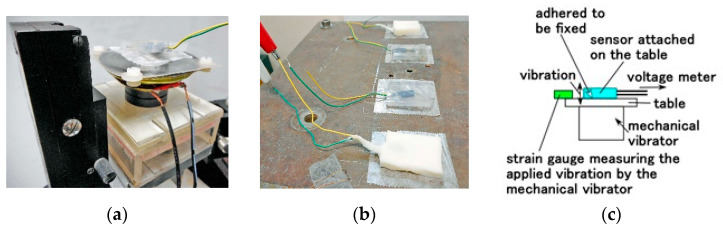
The experimental apparatus: (**a**) equipment for electric vibration; (**b**) equipment for mechanical vibration; (**c**) schematic diagram of (**b**).

When a high-frequency range vibration was applied to the fabricated sensor with an electrical vibrator, we measured the voltage self-generated by the sensor using a voltage meter (PC710, Sanwa Electric Instrument Co., Ltd., Tokyo, Japan). The electrical vibrator demonstrated vibration with a wide frequency, typical of the high-frequency range, utilizing a 0.5 mm-thin soft membrane with a Young’s modulus of 3619 GPa. It was made of Q-rubber solidified with silicon oil (KE1300T) and attached to the cone of a speaker to which vibration was applied vertically with a few mm amplitude by PC through the medium of a rigid acrylic resin pipe, as shown in Figure 2a. The fabricated sensor attached to the Q-rubber is vibrated by the cone generated with an electric signal, whose measurement procedure has been presented in our previous study. The experimental demonstration consisted of a speaker corresponding to the elucidation of an acoustic response, which is another engineering application of high vibration.

On the other hand, another engineering application of automobile and seismic sensing corresponds to low-frequency vibration, as demonstrated with experimental apparatus using a mechanical vibrator (Matsudaira vibrator, UBC-4A, Itoh Seiki Co., Ltd., Tokyo, Japan), with the sensor attached as shown in Figure 2b,c. We measured the self-generated voltage of the sensor using a voltage meter (PC710) and the applied vibration using a strain gauge.

In order to investigate the dynamic property between compressive rate and pressure on the outer cover of the sensor, we used a compression testing machine (SL-6002; IMADA-SS Co., Ltd., Toyohashi, Japan). We measured the self-generated voltage of the sensor with a voltage meter (PC710), whose measurement procedure has been presented in our previous study.

## 4. Results and Discussion

### 4.1. High-Frequency Response

**Figure 3:**
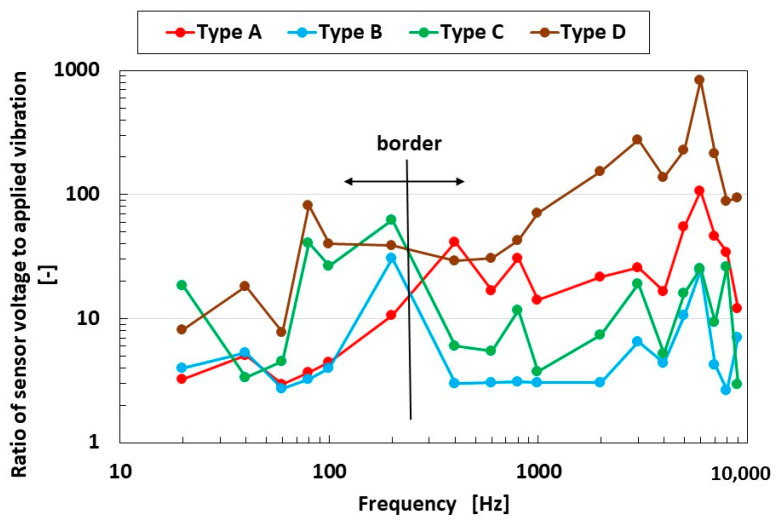
The vibration response of sensor types at a wide range of frequencies due to mechanical vibration.

The experimental demonstration in this section presents the results of the mechanical vibration predominantly for high frequency using the instrument shown in Figure 2a. The ratio of the voltage from the sensor to the applied vibration is arranged by dB level using the first Fourier transform (FFT analysis), as shown in Figure 3.

As may be seen in the figure, on the border between around 200 and 300 Hz, at the frequency less than the border, the ratio of Types C and D was larger than those of Types A and B; in contrast, at higher frequency, the ratio of Types A and D was larger than those of Types B and C. In other words, the Type D sensor with hairs and a U-rubber outer cover was more sensitive under a wide-frequency range than the Type B sensor without hairs and a U-rubber outer cover. Meanwhile, the Type C sensor without hairs and Q-rubber outer cover was more sensitive at the low-frequency range, and the Type A sensor with hairs and Q-rubber outer cover was more sensitive at the high-frequency range. Thus, the results depended on the presence of the hairs and the hardness of the material of the outer cover.

**Figure 4:**
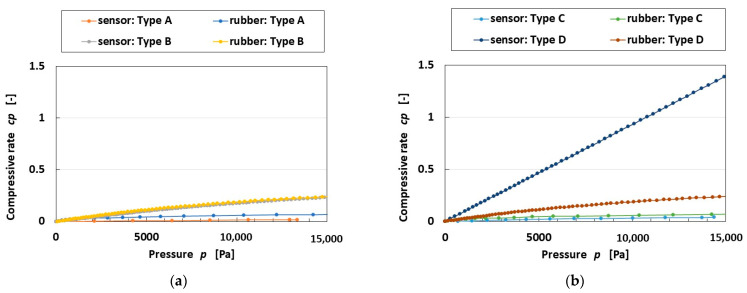
Compressive rate, which means compressive strain rate, to pressure by the sensor type and outer cover: (**a**) Types A and B; (**b**) Types C and D.

We then examined the effect of the hardness of the outer cover by measuring the compression on the sensor and the outer body with the compression testing machine. The results are as presented in Figure 4a,b and Table 2, indicating the quantitative comparison, the approximate curve, and the coefficient of determination: the compressive rate of Q-rubber was less than that of U-rubber because of its greater hardness; on the other hand, when we examined the difference in sensors with and without hairs, the hardness in the case of the Q-rubber outer cover did not differ, while the U-rubber did. We hypothesize the following reason. The difference in the case of U-rubber was due to the chemical conjunction between the HF rubber and U-rubber at the hairs, such that it enhanced the hardness. Therefore, the ratio of the Type D sensor with hairs and U-rubber outer cover was the most sensitive under the wide-frequency range. On the basis that the coefficient of the second term of the approximate curve demonstrates the quantitative tendency of the mean value, a large coefficient of the second term predominantly corresponds to a large quantitative tendency of the relation between the compressive rate and the pressure. A large coefficient of determination corresponds to a large compressive rate of pressure.

**Figure 5:**
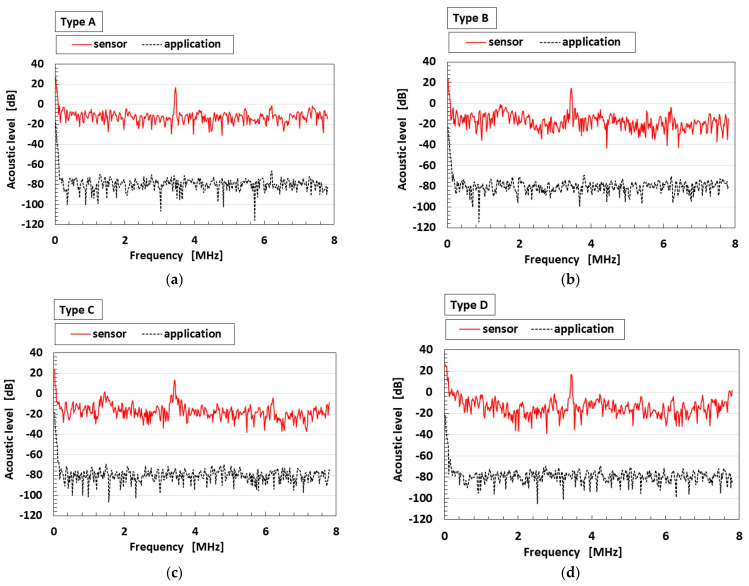
Acoustic level according to sensor type (**a**–**d**).

**Figure 6:**
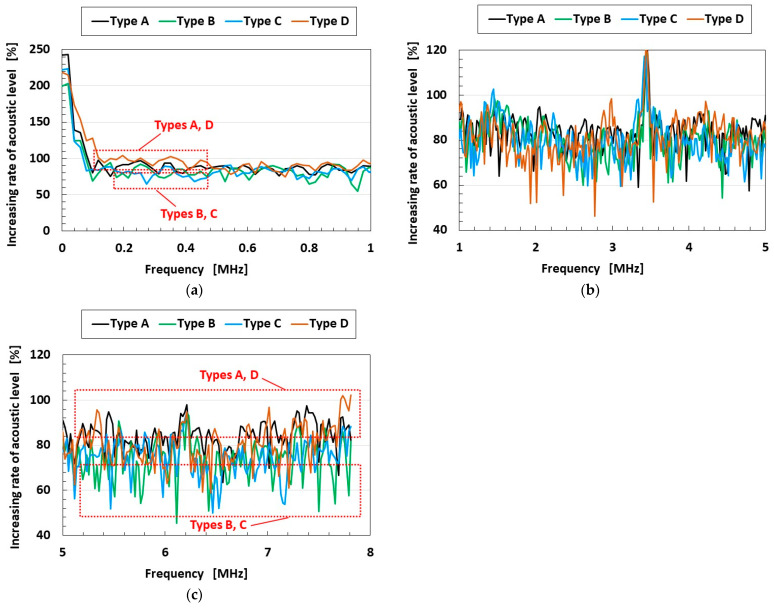
Increasing rate of the acoustic level according to frequency range: (**a**) at 0–1 MHz; (**b**) at 1–5 MHz; (**c**) at 5–8 MHz.

As another example of an engineering application for vibration with high frequency, auditory sensation systems might be applicable for biological and anatomical simulation that mimics the human ear, whose systems include auditory receptors at hair cells in the utricle and at the organ of Corti [28]. When acoustic sound is applied to the sensor using the electric vibration of the speaker in Figure 2a, the response of the sensor is shown in Figure 5a–d. Because the acoustic level of the application in the figure is a sound such as a pop song, classical music, etc., it is assembled from the vibration from the speaker having each frequency from the low to high frequency range by using the instrument as shown in Figure 2a. The acoustic level of the application has many vibrating waves with varied frequencies, which are played in succession. Therefore, the acoustic level of the sensor is presented in response to the application having each frequency. The figure does not show the anomalies of the sensor. As may be seen, the acoustic level of the sensor increases according to each frequency range of applied acoustic sound, as shown in Figure 6a–c. Figure 6 presents the same response of the sensor to the application of a sound composed of many frequencies, as in Figure 5. The increasing rate of Types A and D is greater than that of Types B and C—a result that coincides with the one in Figure 3.

### 4.2. Low-Frequency Response

**Figure 7:**
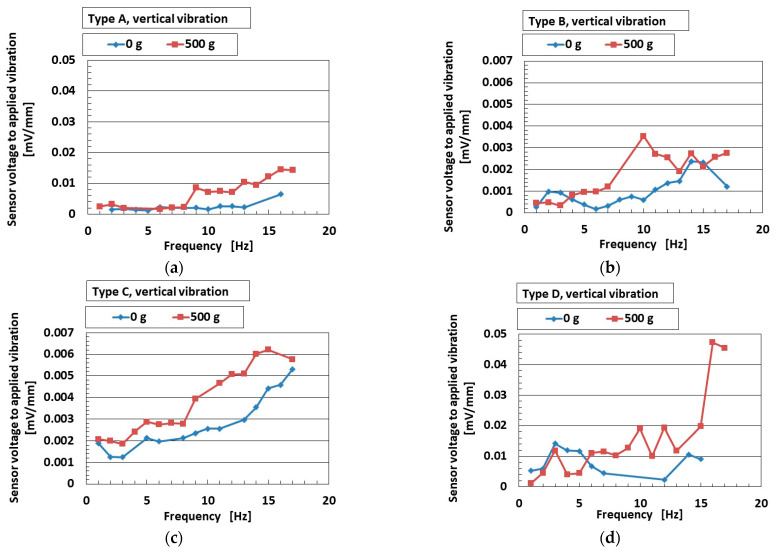
The ratio of the voltage of the sensor to the amplitude of the applied vertical vibration in the low-frequency range: (**a**) Type A; (**b**) Type B; (**c**) Type C; (**d**) Type D.

**Figure 8:**
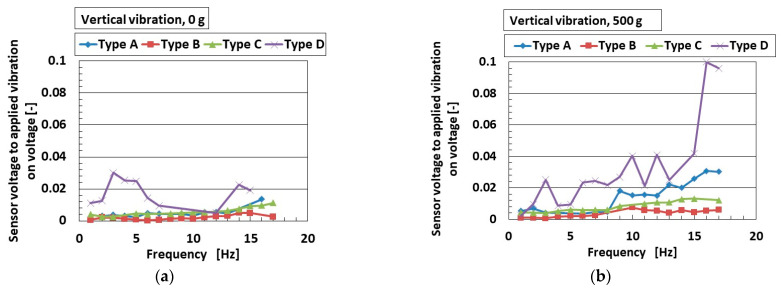
Increasing rate of acoustic level according to frequency range for vertical vibration: (**a**) 0 g; (**b**) 500 g.

**Figure 9:**
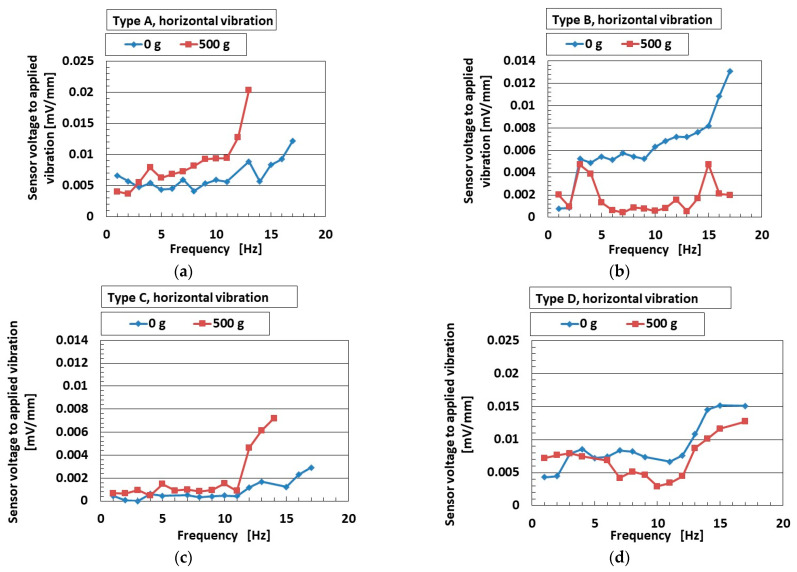
The ratio of the voltage of the sensor to the amplitude of the applied horizontal vibration in the low-frequency range: (**a**) Type A; (**b**) Type B; (**c**) Type C; (**d**) Type D.

**Figure 10:**
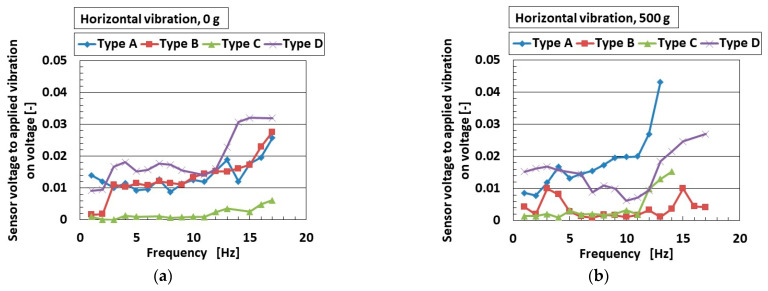
Increasing rate of acoustic level according to frequency range for horizontal vibration: (**a**) 0 g; (**b**) 500 g.

A typical example of an engineering application for low-frequency vibration is found in automobiles and earthquakes, with frequencies lower than around 10–15 Hz. Experimental apparatus with a low-frequency range is thus optimal when using the mechanical vibration implementation as shown in Figure 2b. Figure 7a–d and Figure 8a,b show the ratio of the voltage of the sensor to the applied vibration of the 0.64 mm amplitude for vertical vibration, and Figure 9a–d and Figure 10a,b show the same for the horizontal vibration. In addition, we also present a situation in which a 500 g mass was adhered to the sensor, which could predict the mounting of any bodies during installation in automobiles, etc.

For both vertical and horizontal vibration, the sensitivity of the sensor to the vibration in Type D was the largest of all sensor types, which agrees with the results in Figure 3. On the other hand, the quantitative value of the sensitivity of the sensor in Types A and D was larger than that of Types B and C. We consider that this was due to the presence of the hairs, which increased the sensitivity of the sensors. In contrast, regarding the effect of mass, it enhanced the sensitivity of the sensor to vibration. The largest change in sensitivity by mass was seen in Type D for vertical vibration; in contrast, this was true for Type A for horizontal vibration. This result was due to the different material of the outer cover.

Although the value at the ordinate seems to be similar over the frequency range, as shown in Figure 3, it is clearly different by the kind of sensor Types A–D at the frequency more than the border shown in the figure. In contrast, a frequency lower than the border means that the low frequency range becomes quantitatively ambiguous. However, at the lowest frequency range, the ratio of the voltage of the sensor to the amplitude of the applied vibration is different in terms of the kind of sensor (Types A–D), as shown in Figure 8. Furthermore, the sensor needs to be improved. Our fabricated sensor in the present study had the configuration mimicking the free nerve endings in the human cutaneous receptors. We have already developed other fabrications mimicking other cutaneous receptors in other papers. Therefore, in case of using the other fabrications, the ratio of the voltage of the sensor to the amplitude of the applied vibration could be clearly obtained with a quantitatively large difference so that the fabricated sensor suitably conforms to the practical applications in low frequency.

### 4.3. Multi-Faceted Consideration

In this section, we consider the results of the previous section from a different perspective, essentially treating the stimuli to the vibration, such as firing rate and gauge factor (GF), as in piezo-resistivity, as well as in response performance. The vibration properties in piezo-electricity can be evaluated in terms of how they affect other parameters used in different fields, such as firing rate and GF.

#### 4.3.1. Stimuli to Vibration

The response time of the sensor is an attractive subject. That of the HF rubber is highly sensitive with an msec order. It corresponds to the stimulus of the vibration. The response of the sensor to the applied stimuli in the low-frequency range is more significant than in the high-frequency range because the period of the cycle of the sensor’s voltage is equivalent to one of the low-frequency range vibrations, in which the frequency of the vibration is less than the natural frequency of the sensor. Awareness of stimuli to vibration is significant in various vibration sensors. The voltage of the sensor is then measured through the repeated compression of the sensor at a pressing speed of 5 mm/min using the compression testing machine. The response of the sensor to the stimuli might be evaluated using the firing rate if the sensor is mimicking a biological function, such as the cutaneous receptors utilized in haptic robotics, skin, and devices with artificial mechanoreceptors. As discussed in our previous study, the firing rate is the differentiation of the mean spike count of the voltage of the sensor. This is shown in Equation (1), where λ(*t*) is the firing rate, *N* (*t*) is the mean spike count, and ε is the diminutive increment of *t*. Thus, the firing rate can be estimated as the gradient of the changing voltage, approximating the evaluated equivalent firing rate (e.f.r.). The e.f.r. might also be evaluated as the response time of the sensor.

(1) $$\lambda (t)=\underset{\varepsilon \to 0}{\lim}\frac{N(t,t+\varepsilon )}{\varepsilon }$$

**Figure 11:**
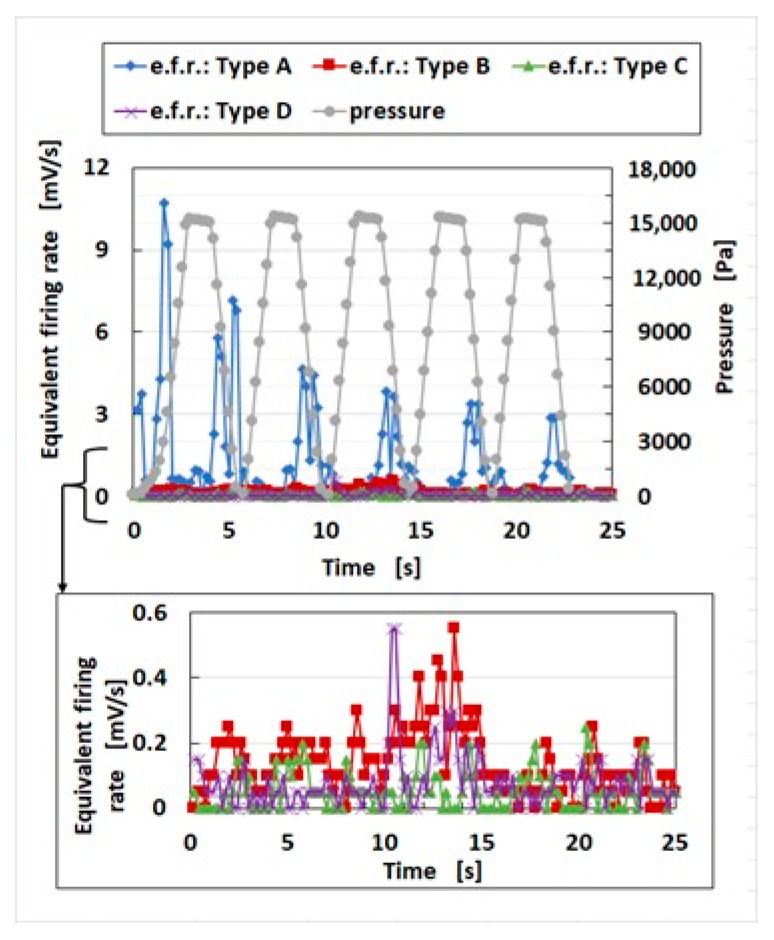
Equivalent firing rate (e.f.r.) of Types A–D and the response time of the sensor.

Figure 11 shows the e.f.r. of sensor Types A–D. The e.f.r. of Type A is the largest of all types; therefore, the sensor might have been the most sensitive to the gradient of the pressure. Consequently, the sensor with hairs and a hard outer cover is the most responsive. Our fabricated sensor can be categorized in FAI, where the fast adaptation (FA) has a typical peculiarity with a high peak at the early stage of the period, which connotes high sensitivity.

Regarding long-term stability, it is not so good in the lowest frequency range, as shown in Figure 11. However, as shown in Figure 3, the response of the sensor to the applied vibration is good in the high frequency range, at least enough to be discriminated quantitatively compared to the low frequency range. In addition, the long-term stability, which means repeatability, is satisfactory in the high frequency range as shown in the next table. Our sensor, fabricated in the present study, displays effectiveness in the high-frequency range. In case our fabricated sensor is adapted in the low frequency range, it is proposed to be improved through the method of utilizing the fabrication with the other mimicked cutaneous receptor as described in Figure 10.

As the performance of the sensor fabricated in the present study—with comparison to other typical stretchable strain sensors (S.S.S.) for the human motion monitoring [29]—has the expectable specificity of state-of-the-art vibratory sensors, Table 3 shows the optimal vibrational performance, which involves the frequency response range, minimum detection frequency, repeatability, and duration, which connotes long-term stability. The minimum detection frequency is involved in the smallest value at the frequency response range, and repeatability is found in the reciprocal nature of the response frequency. In addition, regarding sensitivity and response time, Table 4 presents a comparison to other typical sensors. Incidentally, regarding recovery time, Type A has 50 ms, Type B 2000 ms, Type C 50 ms, and Type D 300 ms. Our fabricated sensor has the peculiarity that the recovery time is smaller than the response time. This sensor is suitable for the high-frequency range, so that the number of vibrations is large enough to be durable. Moreover, it has low sensitivity at a low frequency range but a fast response at a high frequency range. As described in Figure 11, our fabricated sensor displays the effectiveness in the high-frequency range. In the case of adapting this one in the low frequency range, it is proposed to be improved by utilizing the fabrication with the other mimicked cutaneous receptor.

#### 4.3.2. Gauge Factor for Piezo-Electricity

**Figure 12:**
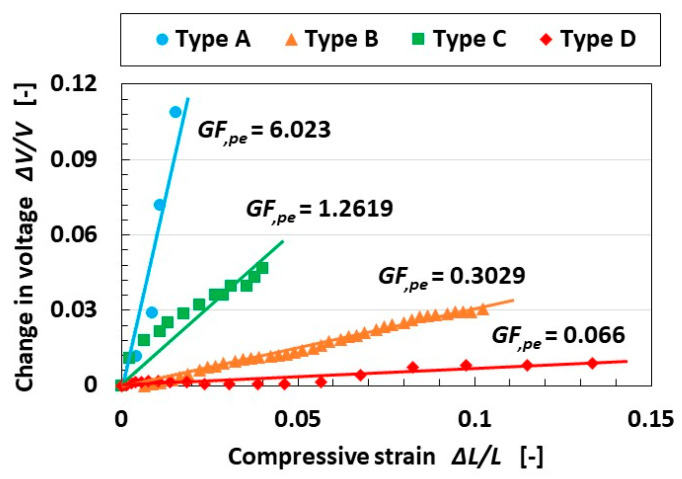
Gauge factor in the case of piezo-electricity relevant to the gauge factor in the case of piezo-resistivity.

In general, in order to evaluate the efficacy of a sensor based on piezo-resistivity, the *GF* is defined by the ratio of the enhancement of the resistance to the strain [50]. Meanwhile, *GF* has not been defined in the case of piezo-electricity. However, in the case of piezo-resistivity, resistance is measured by the voltage of the resistance in a parallel circuit connected to the sensor. The principle of measuring *GF* is relevant to the evaluation of the change in the voltage; therefore, the change in the voltage of piezo-electricity could be related to *GF*. As a result, it can be denoted that the *GF* of piezo-electricity might be defined by the same means as the *GF* in piezo-resistivity, as shown in Equation (2). We denote this as *GF*_,*pe*_, as shown in Figure 12. The values were obtained from the measurement of the voltage of the sensor using the compression testing machine. Here, Δ*V* is the difference in the voltage of the sensor by compression, *V*_0_ is the initial voltage, Δ*L* is the difference in compression, and *L*_0_ is the initial thickness of the sensor.

(2) $${GF}_{,pe}=\frac{∆V/V_{0}}{∆L/L_{0}}$$

The results indicate that the *GF*_,*pe*_ of Type A was the largest of all types—the same result as in Figure 11. Therefore, the sensor with hairs and a hard outer cover not only has the largest change in the voltage of the sensor by compressive strain in evaluating *GF*_,*pe*_, but is as highly responsive as e.f.r. However, those results do not always correspond to the vibration results, as shown in Figure 7, Figure 8, Figure 9 and Figure 10, where the sensor voltage to the applied vibration in Type D was the largest.

In reference to the *GF* of the other piezo-resistivity sensor, the auxetic metamaterial stretchable strain sensor has a 1–10-ordered *GF* [51]; the sensor with CNT mimicking Ruffini corpuscles as a mechanoreceptor has a 10–20-ordered *GF* [29]. The stretchable sensor is made with conductive thermoplastic CNT and a PDMS 0.1-ordered *GF* [52], and the wearable flexible sensor is made with CNT, superhydrophobic conductive rubber, and SiO_2_ not more than 700 *GF* [53]. We might guess that, at present, our sensor is almost equivalent in response to the other piezo-resistivity sensor. Incidentally, *GF* in the case of bare MCF rubber, which is improved before the HF rubber without covering, is 0.005–0.05, and the HF rubber has the same order of *GF*.

## 5. Conclusions

The results of the voltage from the sensor to the applied vibration, performed using the mechanical vibration equipment for high frequency, depend on the presence of hairs in the sensor and the hard material of its outer cover. The same results could be obtained in auditory sensation systems as another example of an engineering application of vibration with high frequency, such as the acoustic sound of a speaker. On the other hand, the results in low frequency with mechanical vibration equipment were more complicated. The other examples of low-frequency vibrating engineering applications include automobiles and earthquakes. Our fabricated sensor is applicable for a wide range of frequencies (1–10,000 Hz) and predominantly performs optimally in the high frequency range.

From a different perspective, we proposed two parameters, stimuli to the vibration as the equivalent firing rate (e.f.r.), and gauge factor (*GF*_,*pe*_), which are currently treated in other engineering fields, and were effective enough to aid in the comprehension of the experimental results in the present study. That is, evaluation using these parameters was effective enough to design a sensor based on piezo-electricity. The e.f.r. is enhanced by the hairs in the sensor and the hardness of the outer cover, which performs in the *GF*_,*pe*_.

These results are effective enough to be useful in various vibration engineering fields, including industry, surgery, space, and mechanics, such as remote and autonomous driving. Thus, mechanoreceptors that mimic the human cutaneous receptor could be applicable in the engineering application of vibratory sensing, because the performance corresponds to the haptic case in which vibration can be perceived by the human hand touching a vibrating body. The configuration of the sensor with the mechanoreceptor is different from current vibration sensors, such as strain gauges, piezo materials, etc. Moreover, the proposed vibration sensor is a novel and cutting-edge approach to vibration sensing.

## Figures and Tables

**Table 1:** Ingredients of HF rubber for the fabrication of the receptors.

Ingredients	HF Rubber 1	HF Rubber 2	HF Rubber 3	HF Rubber 4
water	3 g	3 g	1 g	1 g
sodium tungstate (VI) dehydrate (Na_2_WO_4_ 2H_2_O, Fujifilm Wako Chemical Co., Ltd., Osaka, Japan)	0.5 g	0.5 g	-	0.5 g
TiO_2_	0.5 g	0.5 g	0.5 g	0.5 g
HF	1 g	1 g	1 g	1 g
NR-latex (Ulacol; Rejitex Co., Ltd., Atsugi, Japan)	3 g	3 g	3 g	3 g
CR-latex (671A; Showa Denko Co., Ltd., Tokyo, Japan)	3 g	3 g	3 g	3 g
carbonyl Ni powder (BASF Japan Co., Ltd., Tokyo, Japan)	3 g	3 g	3 g	3 g

**Table 2:** Summary of the results of Figure 4.

Type of Sensor and Rubber	Quantitative Comparison	Approximate Curve	Coefficient of Determination
sensor: Type A	low	*cp* = −2 × 10^−11^ *p*^2^ + 2 × 10^−6^ *p*	0.9892
rubber: Type A	low	*cp* = −6 × 10^−11^ *p*^2^ + 5 × 10^−6^ *p*	0.9477
sensor: Type B	middle	*cp* = −4 × 10^−10^ *p*^2^ + 2 × 10^−5^ *p*	0.9997
rubber: Type B	middle	*cp* = −3 × 10^−10^ *p*^2^ + 2 × 10^−5^ *p*	0.9934
sensor: Type C	low	*cp* = −1 × 10^−10^ *p*^2^ + 5 × 10^−6^ *p*	0.9958
rubber: Type C	low	*cp* = −6 × 10^−11^ *p*^2^ + 5 × 10^−6^ *p*	0.9477
sensor: Type D	high	*cp* = −3 × 10^−23^ *p*^2^ + 9 × 10^−5^ *p*	1
rubber: Type D	middle	*cp* = −3 × 10^−10^ *p*^2^ + 2 × 10^−5^ *p*	0.9932

**Table 3:** The optimal vibrational performance and duration of the sensor fabricated in the present study, with comparisons to other typical S.S.S., whose detailed configuration has been presented in the reference.

Kinds of Sensors with Material	Optimal Vibrational Performance [Hz]	Duration [cycles]
S.S.S. with carbon nanotube (CNT), carbon black (CB), polyurethane, and polydimethylsiloxane (PDMS) [30]	0.083	2500
S.S.S. with graphene and PDMS [31]	70	36,000
S.S.S. with CNT and biodegradable plastic (Ecoflex) [32]	0.067	2000
S.S.S. with multiwalled CNT (MWCNT) and PDMS [33]	0.067	1000
S.S.S. with PVA, PDMS, and Ag nano-particles (AgNP) [34]	100	2000
S.S.S. with vertically aligned carbon nanotube (VACNT) and Ecoflex [35]	4	10,000
S.S.S. with CNT, Ag nano-wire (AgNW), and thermoplastic polyurethane pellets (TPUs) [36]	2	2500
S.S.S. with CB, graphene, and Ecoflex [37]	1	4000
S.S.S. with MWCNT and Ecoflex [29]	40	12,000
present sensor in this study (Types A–D)	200–10,000	10,000

**Table 4:** The performance of sensitivity and response time of the sensor fabricated in the present study, with comparison to other typical sensors, whose detailed configuration has been presented in the reference.

Kinds of Sensors with Material	Sensitivity [mV/Pa]	Response Time [ms]
triboelectric sensor [38]	1.06	
triboelectric nanogenerator (TENG) [39]	3.4	
polyvinylidene difluoride (PVDF)-based triboelectric film [40]	1.48	
triboelectric capacitive-coupled tactile sensor [41]	7.88	
piezoelectric energy harvesters (PEHs) [42]	0.29	
triboelectric active pressure sensor [43]	12.8	
flexible capacitive sensor based on Miura-ori structure [44]		100
flexible pressure sensor with thermoplastic microspheres (TPM) [45]		98
high anti-jamming capacitive flexible pressure sensor with PVD, AgNWs, and TiO_2_ film [46]		166.9
capacitive tactile sensors with cone-shaped electrodes [47]		100
flexible capacitive sensors with template-free formation of hybrid dielectric [48]		97
skin-inspired capacitive flexible tactile sensor [49]		60
present sensor in this study (Type A at low frequency)	4.4 × 10^−4^	100
present sensor in this study (Type B at low frequency)	1.8 × 10^−4^	5000
present sensor in this study (Type C at low frequency)	9.5 × 10^−6^	100
present sensor in this study (Type D at low frequency)	5.1 × 10^−5^	600
present sensor in this study (Types A–D at high frequency)		0.1

## Data Availability

The original contributions presented in this study are included in the article. Further inquiries can be directed to the author.
